# Heterogeneity of miRNA expression in localized prostate cancer with clinicopathological correlations

**DOI:** 10.1371/journal.pone.0179113

**Published:** 2017-06-19

**Authors:** Ahmed Hussein Zedan, Søren Garm Blavnsfeldt, Torben Frøstrup Hansen, Boye Schnack Nielsen, Niels Marcussen, Mindaugas Pleckaitis, Palle Jörn Sloth Osther, Flemming Brandt Sørensen

**Affiliations:** 1 Urological Research Center, Department of Urology, Vejle Hospital, Vejle, Denmark; 2 Department of Oncology, Vejle Hospital, Vejle, Denmark; 3 Institute of Regional Health Research, University of Southern Denmark, Odense, Denmark; 4 Bioneer A/S, Hørsholm, Denmark; 5 Department of Pathology, Odense University Hospital, Odense, Denmark; 6 Department of Clinical Pathology, Vejle Hospital, Vejle, Denmark; Universite Clermont Auvergne, FRANCE

## Abstract

**Introduction:**

In the last decade microRNAs (miRNAs) have been widely investigated in prostate cancer (PCa) and have shown to be promising biomarkers in diagnostic, prognostic and predictive settings. However, tumor heterogeneity may influence miRNA expression. The aims of this study were to assess the impact of tumor heterogeneity, as demonstrated by a panel of selected miRNAs in PCa, and to correlate miRNA expression with risk profile and patient outcome.

**Material and methods:**

Prostatectomy specimens and matched, preoperative needle biopsies from a retrospective cohort of 49 patients, who underwent curatively intended surgery for localized PCa, were investigated with a panel of 6 miRNAs (miRNA-21, miRNA-34a, miRNA-125b, miRNA-126, miRNA-143, and miRNA-145) using tissue micro-array (TMA) and *in situ* hybridization (ISH). Inter- and intra-patient variation was assessed using intra-class correlation (ICC).

**Results:**

Four miRNAs (miRNA-21, miRNA-34a, miRNA-125, and miRNA-126) were significantly upregulated in PCa compared to benign prostatic hyperplasia (BPH), and except for miRNA-21 these miRNAs documented a positive correlation between the expression level in PCa cores and their matched BPH cores, (*r* > 0.72). The ICC varied from 0.451 to 0.764, with miRNA-34a showing an intra-tumoral heterogeneity accounting for less than 50% of the total variation. Regarding clinicopathological outcomes, only miRNA-143 showed potential as a prognostic marker with a higher expression correlating with longer relapse-free survival (*p* = 0.016).

**Conclusion:**

The present study documents significant upregulation of the expression of miRNA-21, miRNA-34a, miRNA-125, and miRNA-126 in PCa compared to BPH and suggests a possible prognostic value associated with the expression of miRNA-143. The results, however, document intra-tumoral heterogeneity in the expression of various miRNAs calling for caution when using these tumor tissue biomarkers in prognostic and predictive settings.

## Introduction

Prostate cancer (PCa) is the leading cancer among men and the second most common cause of cancer related death in men in the Western World [1–3]. The techniques for diagnosing PCa have evolved over the past decades and encompass prostate specific antigen (PSA), digital rectal examination and histological Gleason score (GS) of needle biopsies. Although diagnostics have improved the management of the disease, there are still concerns about the specificity and sensitivity of these diagnostic tools [4–6].

Needle biopsy GS has been confirmed to be one of the most important prognostic factors of PCa, whereas it remains a poor predictor of clinical outcome [7]. Introducing a histological malignancy score (GS) in 1966, *D*. *Gleason* recognized the impact of heterogeneity on GS. Accordingly, he introduced the primary and secondary histologic pattern[8], when evaluating PCa for obtaining the GS. Moreover, discrepancy between needle biopsy GS and overall GS of radical prostatectomy (RP) specimens is a well-known issue, which may influence the clinical outcome after surgical intervention [9]. Histological discrepancy regarding GS can be demonstrated in approximately half of patients undergoing surgery for localized PCa, with the vast majority experiencing an upgrading of GS in their surgical specimen [10]. About 80% of prostatectomy specimens harbor more than one cancer focus that may expose different GS [11,12]. In addition, the histological GS obtained in the diagnostic needle biopsy is even more critical for non-surgical options such as active surveillance, watchful waiting, or radiotherapy, where the biopsy is the only source of tumor tissue for grading. Among the possible factors contributing to this inconsistency are varying reproducibility of the GS [13], and most importantly, sampling error due to a limited number of needle biopsies with varying degree of cancer infiltration [14]. Moreover, recent research has disclosed that adoption of the new International Society of Urological Pathology (ISUP) grades [15] would increase the discordance between GS as scored in the needle biopsy and RP [16].

Accordingly, there is an urgent need to define more reliable biomarkers in diagnostic, prognostic and predictive settings that may contribute to solving these clinical challenges.

Investigating microRNA (miRNA) may offer an approach towards this goal. Since their discovery more than two decades ago miRNAs have attracted considerable attention in cancer biology, as their expression may reflect the transitional process of malignancy [17]. MiRNAs are evolutionarily conserved, non-coding single-stranded RNA molecules comprising 19–24 nucleotides that regulate gene expression at different levels [18]. MiRNAs have been associated with various cellular processes such as development [19], differentiation [20], cell cycle regulation, cell cycle arrest [21], and apoptosis [22]. Additionally, several studies have demonstrated miRNAs as useful biomarkers for cancer diagnosis, prognosis, prediction of treatment efficacy, and even as possible therapeutics in PCa [23–25].

In this methodological study we selected a series of miRNAs for investigation, *i*.*e*. miRNA-21, miRNA-34a, miRNA-125b, miRNA-126, miRNA-143, and miRNA-145, which have been reported in PCa biology (S1 Table) aiming at defining *in situ* expression, heterogeneity and correlation with clinicopathological characteristics in localized PCa.

## Material and methods

### Patients

This retrospective investigation is based on a cohort of 53 patients diagnosed with localized PCa (according to the UICC 2002 TNM system [26]), who underwent curatively intended radical retropubic prostatectomy (RRP) and regional lymph node dissection in the period between April 2004 and March 2006 at the Department of Urology, Frederica Hospital, Denmark. Three patients were excluded from the tissue microarray analysis (TMA), and two patients from the needle biopsy analysis (one of which was also excluded from the TMA analysis), due to insufficient archival tissue left in the formalin-fixed, paraffin-embedded (FFPE) tissue blocks leaving 49 patients for analyses. One patient was treated with neoadjuvant LHRH-agonist (Goserelin) and anti-androgen (Cyproterone). Four patients had received a 5-alpha reductase inhibitor (5ARI) (Finasteride) during their management and before RRP. Despite the ongoing discussion about the use of 5ARI and increased risk of high GS, there is still not enough scientific data to support this hypothesis [27]. These five patients were not excluded from the study cohort due to the methodological scope of the present investigation.

Stratification of PCa patients according to risk of recurrence was based on both the *National Comprehensive Cancer Network* (NCCN) guidelines for PCa [28] and the *D’Amico* classification [29].

The study was approved by The Regional Committees on Health Research Ethics for Southern Denmark (S-20140201) according to Danish law. The Committee waived the need for patient consent to the use of the archived tissue samples. In addition, the Danish Data Agency approved the study and the Danish Registry of Tissue Utilization was screened prior to study initiation.

### Clinical data

The pre-surgical PSA level was defined as the last measurement prior to transrectal ultrasound. All patients had follow-up with serum PSA every three months for the first two years, every six months the following three years, and annually after the fifth post-operative year. In non-relapsing patients the follow-up PSA was defined as the most recent measurement, whereas in the case of relapse, the PSA measurement at the time of biochemical recurrence (BCR) was used. BCR was defined as two consecutive PSA values of 0.2 ng/ml or higher and rising [30].

### Tissue

All FFPE tissue samples from needle biopsies as well as RRP specimens had been routinely processed for pathoanatomic diagnosis and were retrieved from the archives of the Department of Clinical Pathology, Vejle Hospital, Denmark. In most cases, six needle biopsies were available from each patient, whereas all tissue from the RRP had been embedded at the original tissue processing. Four μm thick, hematoxylin-eosin (H&E) stained routine sections from all tissue blocks were reviewed by experienced uropathologists. The needle biopsy showing the highest GS was selected using the updated GS from 2005 [31]. The same approach was implemented for the RRP specimens, selecting 2–4 tissue blocks with the highest GS and one with benign prostatic hyperplasia (BPH) to be further processed for TMA.

### Construction of tissue microarray

Before constructing the TMA, one H&E stained tissue section was cut from each of the selected tissue blocks to obtain morphologically representative areas with BPH and PCa showing the highest GS. The TMA was produced using the Advanced Tissue Arrayer® (Chemicon International) guided by the highlighted area of the H&E section. Two mm tissue cores were transferred and deposited into pre-manufactured paraffin recipient blocks (Tissue-Tek®, Sakura, The Netherlands). The tissue cores were orientated using one core of placenta tissue serving as a guide for navigating through the TMA coordinate system, thus enabling identification of each core in the whole TMA. After transferring all cores, fusion of the recipient block was achieved by heating the block at 60°C for one hour. Subsequently, 5 μm thick tissue sections were cut from the TMAs, and the first section was H&E stained to verify the representation of tissue and identify lost cores in the section. Furthermore, sections were used for *in situ* hybridization (ISH). After verification of lost tissue cores, the TMA technique produced two (N = 6), three (N = 15) or four (N = 28) tissue cores with PCa and one core with BPH from each patient.

### In situ hybridization

ISH was performed essentially as described previously [32]. In brief, the ISH analysis was performed on 5 μm thick sections using a Tecan Evo instrument™ (Männedorf, Switzerland). To determine optimal probe concentrations and hybridisation temperatures, the assay parameters were evaluated prior to the analysis. Sections were pre-digested with proteinase-K (15 μg/ml) at 37°C for 8 minutes, pre-hybridised at 55°C for 15 minutes, and hybridised with double-carboxyfluorescein (FAM) Labeled Locked Nucleic Acid (LNA) probes [33] (Exiqon A/S, Vedbaek, Denmark) at 55^°^C with miRNA-21 (30nM), miRNA-34a (40nM), miRNA-125b (30nM), miRNA-126 (30nM), miRNA-143 (20nM), miRNA-145 (20nM), or the scramble probe (30nM). After stringent washes in saline-sodium citrate buffer the probes were detected with alkaline phosphatase-conjugated sheep anti-FAM Fab fragments followed by incubation in substrate containing 4-nitroblue tetrazolium and 5-bromo-4-chloro-3’-Indolylphosphate (Roche, Denmark) for 60 minutes, resulting in a dark-blue staining, and finally counterstained with nuclear fast red (Vector Laboratories, CA, USA). The ISH analyses and subsequent image analysis using the Visiopharm TMA module were performed by Bioneer A/S (Hoersholm, Denmark).

To reduce variation related to ISH processing of the slides, all tissue sections were cut on the same day and all sections were stained with alike probes in the same run within a week thereafter. The experimental parameters indicated above used for staining of the individual probes had been optimized on sections from FFBE needle biopsies.

### Image analysis

Image analysis was carried out as a 2-step procedure using the TMA workflow module within the VisiomorphDP software™ (Visiopharm, Denmark). The TMA software tool allows alignment of cores in the TMA and the macro-dissected tumor areas in the needle biopsies into regions of interest (ROI). The TMA sections were stained for the various miRNAs using automation, where the slides were placed under a cover glass forming an “incubation chamber”, and the liquid reagents were retained in the chamber by capillary action. It could not be fully avoided that air bubbles were developing in the incubation chambers during the in situ hybridization staining procedure and caused non-specific NBT-BCIP staining that were easily visible on the tissue during microscopy examination. Also staining artefacts caused by air bubbles and insufficient liquid flow leading to uneven staining were cleared. The un-specifically stained areas were “cleared” manually in the software. All cores were processed with the same pixel classifier (S1 Fig). The image analysis (pixel classification) was designed to discriminate two intensity levels of the ISH signal (weak and intense, abbreviated wB and iB). To compare estimates of stained areas (iB or iB + wB), the total tissue area (TT) and the total red area (TR) (*i*.*e*. a measure of nuclear density) in TMA cores and the total area (AA) in needle biopsies were determined. These area fractions (iB/TT or (iB+wB)/TT) were considered relative miRNA expression estimates. Different signals of miRNA expression were chosen (according to the most reliable expression in the ROIs) to be analyzed (See S2 Table). The entire ISH procedure and following image analysis were performed by an investigator (BSN) unaware of the clinical variables.

### Statistics

The heterogeneity of miRNA expression was investigated using mixed effect linear regression analysis with random effects for clustering at the subject level, and estimates of interclass correlation (ICC) were calculated to evaluate the correlation of miRNA expression among cores taken from individual patients embedded in the TMA. A high ICC reflects a high degree of correlation between cores ***within*** patients (= *intr****a***-patient correlation), corresponding to the total variation being dominated by the variation ***among*** patients (= *inte****r***-patient variation or the biological variation).

The mean miRNA expression in TMA cores with tumor was compared with the miRNA expression in the paired needle biopsy from the same patient, using Spearman’s rho. The same statistics was used for testing the correlation between the mean miRNA expression in TMA cores with tumor and that in matched BPH from the same patient. Up or downregulation of miRNA expression in the TMA cores with tumor (mean values), as compared to the core with BPH, was tested for statistical significance using the Wilcoxon signed-rank test. The levels of miRNA expression were tested for correlation with pre-biopsy PSA, pT-stage, GS in needle biopsies and in RRP specimens, and *D’Amico*/NCCN risk profiles, using Spearman’s rank correlation.

Relapse-Free Survival (RFS) was defined as the time from surgery to BCR or death of any cause. For patients without BCR, the RFS was calculated as the time between surgery and the most recent measurement of PSA. Finally, the miRNA expressions were tested for their independent influence on RFS, using a simple Cox regression analysis. These analyses were adjusted for the delayed time from diagnosis to surgery (= lead time), as some patients were operated within a month, others one year after the diagnosis.

All analyses were performed in STATA version 13 (STATACorp, TX, USA), and all tests were 2-sided with *P*-values less than 0.05 considered statistically significant.

## Results

### Clinicopathological data

The clinicopathological data of the 49 patients included in the study are presented in Table 1.

**Table 1:** Clinicopathological data of 49 patients who have been treated for local PCa who underwent curatively intended retropubic prostatectomy.

	No (%)
49	
**Mean age (Range)-year**	
62.7 (52–71)	
**PSA level**	
< 10	28(57)
10 to 20	17(35)
> 20	4(8)
**GS (NB)**	
≤ 6	19(39)
7	28(57)
≥ 8	2(4)
GS (prostate)*	
≤ 6	1(2)
7	47(96)
≥ 8	1(2)
**pT**	
pT2a	4(8)
pT2b	5(10)
pT2c	32(66)
pT3a	3(6)
pT3b	5(10)
**BCR**	
No	24(49)
Yes	19(39)
Always > 0.1 ng/ml**	6(12)
**Death of any cause**	**7(14)**
**D'Amico risk profile**	
Low	8(16)
Intermediate	32(66)
High	9(18)
**NCCN risk profile**	
Very low	2(4)
Low	6(12)
Intermediate	34(70)
High	7(14)

BCR: Biochemical relapse; GS: Gleason score; NB: Needle biopsy; NCCN: National Comprehensive Cancer Network; PSA: Prostate specific antigen; pT: Pathological T-stage; SD: Standard deviation.

*: The highest GS

**: Patients who had not been radically operated.

### Localization of miRNA expression in prostate tumor samples

**Fig 1:**
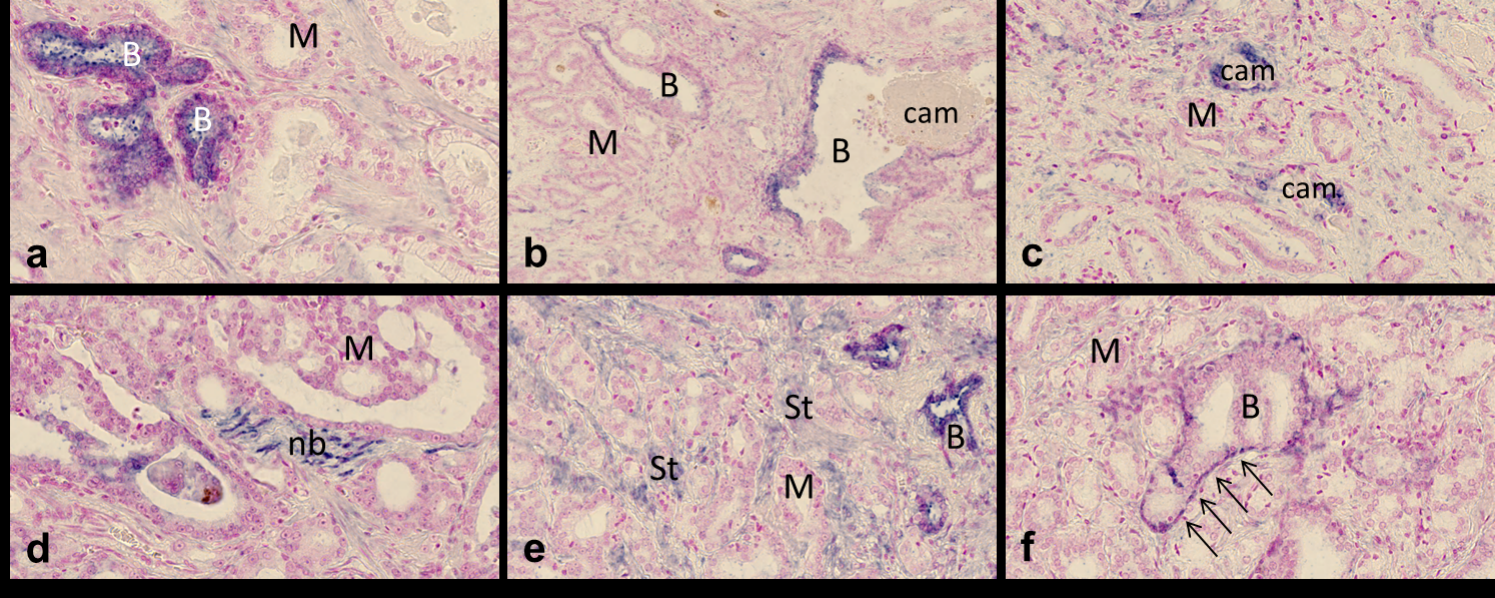
In situ hybridization of miRNA-21 in prostate cancer. **a)** Expression in benign glandular epithelial cells (B), whereas malignant, adenocarcinoma glands (M) are negative; **b)** Expression in benign and **c)** malignant glandular epithelium with luminal corpora amylacea (cam); **d)** Expression in nerve bundles (nb), infiltrated with miRNA-21 negative adenocarcinoma cells; **e)** Expression in tumor stroma (St, myofibroblastic / smooth muscle cells); **f)** Expression in myoepithelial cells (arrows) surrounding benign epithelium. Bars: a, c-f = 40μm, b = 80μm.

**Fig 2:**
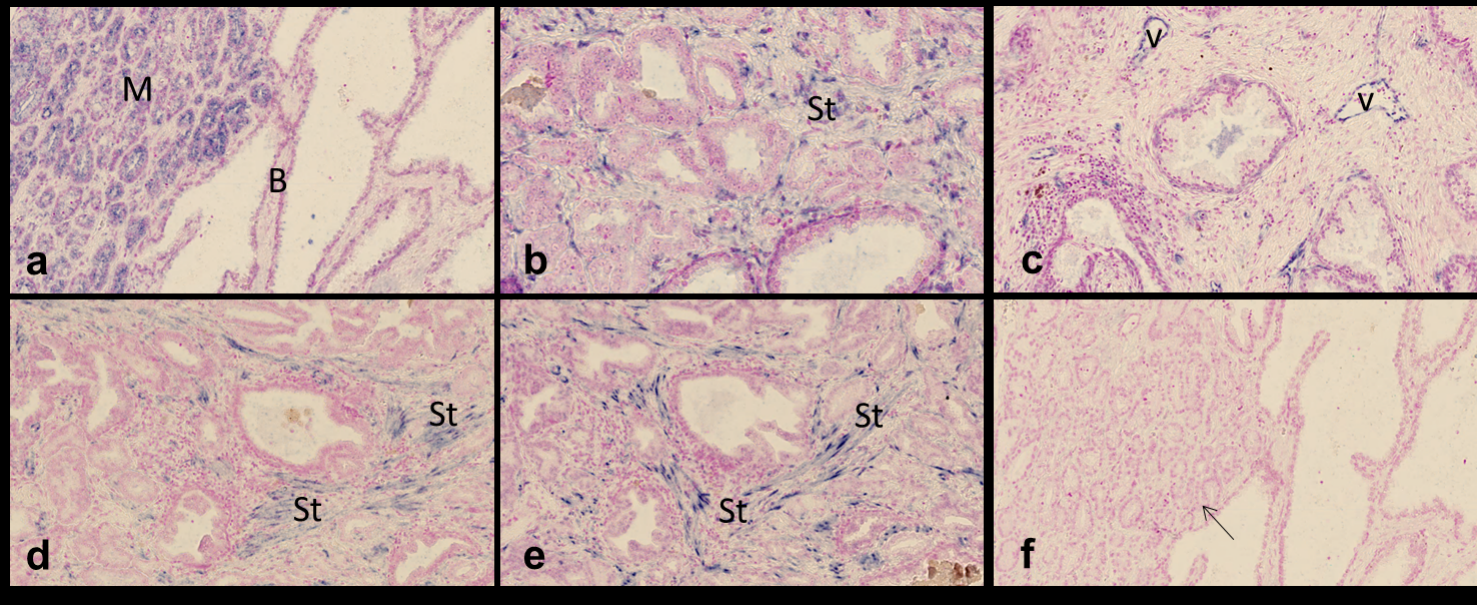
miRNA *in situ* hybridization in prostate cancer. **a)** miRNA-34a expression in malignant, adenocarcinoma cells (M), with no staining in benign epithelial cells (B) of dilated glands; **b)** miRNA125b expression in stromal fibroblastic cells (St); **c)** miRNA-126 expression in endothelial cells of vessels (v); **d)** miRNA-143 expression in stromal smooth muscle cells (St); **e)** Serial section with **d**, showing miRNA-145 expression in stromal cells (St, myofibroblastic/smooth muscle cells); **f)** Serial section with **a**, showing no staining obtained with the scramble probe. Bars: a, c-f = 80μm, b = 40μm.

Examination of the TMA cores indicated different expression patterns of the different miRNAs. The miRNA-21 expression pattern was quite complex (Fig 1). Expression was particularly seen in benign prostate epithelium, whereas malignant epithelium was generally negative. Interestingly, miRNA-21 expression in benign glands was generally seen in the presence of cancer cells, whereas benign glands located in benign surroundings were negative. Both benign and malignant epithelium associated with corpora amylacea was focally positive. Occasionally, miRNA-21 staining was seen in nerve bundles, fibroblastic tumor stroma cells, and myoepithelial cells surrounding benign glands. MiRNA ISH analyses also identified expression of miRNA-34a, miRNA-125b, miRNA-126, miRNA-143, and miRNA-145 (Fig 2). MiRNA-34a was particularly seen in epithelium, whereas miRNA-125b was restricted to fibroblastic tumor stroma cells. As expected, miRNA-126 was seen in vessels and miRNA-143 and miRNA-145 in stromal smooth muscle cells. The cellular origin of miRNA expression is summarized in S3 Table.

The ISH miRNA-expression staining pattern varied in intensity from negative to intensely positive (S1 Fig). This variation is reflected in the quantitative image analyses data obtained from the samples cores.

### Heterogeneity of miRNA expression in PCa

The quantitative expression estimates obtained by image analysis of the ISH signals in the TMA cancer cores showed wide intra-patient correlation for the various miRNAs (S1 Dataset). As shown in Table 2, the highest intra-class correlation was obtained for miRNA-34a with ICC = 0.764 (std. error = 0.051; 95% CI = 0.650–0.850), while miRNA-125b showed the lowest intra-class correlation with ICC = 0.451 (std. error = 0.093; 95% CI = 0.282–0.631). These data reflect the variation of the miRNA expression among patients, *i*.*e*. for miRNA-34a 76% of the total variation can be explained by the inter-patient variation (= biological variation), whereas for miRNA-125b more than 50% of the total variation can be explained by “measurement noise” (= intra-patient = intra-tumoral heterogeneity).

**Table 2:** Heterogeneity of miRNA expressions in PCa in TMA’s.

miRNA	ICC*	Std. err.	95% CI
miRNA-21	0.525	0.087	0.358–0.686
miRNA-34a	0.764	0.051	0.650–0.850
miRNA-125b	0.451	0.093	0.282–0.631
miRNA-126	0.653	0.068	0.512–0.771
miRNA-143	0.627	0.072	0.478–0.754
miRNA-145	0.722	0.060	0.591–0.823

ICC: interclass correlation

PCa: Prostate cancer, TMA: Tissue microarray

* High ICC means higher correlation between cores within patients, leaving most of the overall variation attributable to differences between patients (= biological variation)

When comparing the miRNA expression in TMA PCa cores with the paired needle biopsy (S2 Dataset), there was a poor correlation varying from *r* = -0.0002 for miRNA-143 to *r* = -0.2494 for miRNA-34a, (Table 3).

**Table 3:** Correlation between miRNA expression (mean value) in PCa cores in TMA and paired needle biopsies.

miRNA	Correlation coefficient*	*p*-value
miRNA-21	-0.080	0.590
miRNA-34a	-0.249	0.084
miRNA-125b	-0.150	0.308
miRNA-126	-0.197	0.179
miRNA-143	-0.0002	0.999
miRNA-145	-0.074	0.613

PCa: Prostate Cancer

TMA: Tissue microarray

*Correlation coefficients are Spearman’s rho

### Expression of miRNAs in PCa and BPH

The expressions of miRNA-21, miRNA-34a, miRNA-125b, and miRNA-126 were significantly upregulated in PCa cores compared to their matched BPH cores (Table 4). While miRNA-126 was more than doubled in the cancer cores, miRNA-34a was only marginally elevated in PCa cores compared to the matched BPH cores. Moreover, all 4 upregulated miRNAs showed a good correlation (*r* > 0.72) between PCa cores and the matched BPH cores, except miRNA-21, which showed a rather poor correlation (*r* = 0.53). (S3 Dataset)

**Table 4:** Correlation analysis between miRNA expressions in cancer cores and the BPH core in TMA of 49 patients, who have been treated for local PCa with curatively intended retropubic prostatectomy.

miRNA		Core	BPH
miRNA-21	mean	0.033	0.023
	SD	0.022	0.013
	*p*-value	0.001	
	*r*	0.529	
miRNA-34a	mean	0.038	0.037
	SD	0.031	0.039
	*p*-value	0.020	
	*r*	0.785	
miRNA-125b	mean	0.0002	0.0001
	SD	0.0002	0.0001
	*p*-value	0.0010	
	*r*	0.721	
miRNA-126	mean	0.001	0.0004
	SD	0.001	0.0005
	*p*-value	0.0000	
	*r*	0.743	
miRNA-143	mean	0.041	0.049
	SD	0.033	0.044
	*p*-value	0.300	
	*r*	0.724	
miRNA-145	mean	0.106	0.125
	SD	0.075	0.086
	*p*-value	0.153	
	*r*	0.728	

BPH: Benign prostate hyperplasia

PCa: Prostate cancer

*p*-value are from Wilcoxon signed-rank test; Correlation coefficients are Spearman’s rho

*r*: Correlation coefficient

SD: Standard deviation

TMA: Tissue microarray

### Comparison between miRNA expression and clinicopathological data

Except for miRNA-125b expression marginally correlated with pT- stage (*p* = 0.042), the ISH signals in cancer tissue of the needle biopsies showed poor correlation of the 6 miRNAs with any of the clinical variables, pre-biopsy PSA, GS in needle biopsies and RRP specimens, pT-stage, and *D’Amico*/NCCN risk profiles (Table 5). Only miRNA-143 was significant regarding prognostic impact (HR = 0.32; 95% CI = 0.12–0.81; *p* = 0.016), indicating a longer RFS with increasing miRNA-143 expression (Table 6). (S4 Dataset)

**Table 5:** Correlation analysis of miRNA expressions in needle biopsies and clinicopathological data of 49 patients, who have been treated for local PCa with curatively intended retropubic prostatectomy.

miRNA	Test	**Prebiopsy PSA**	GS (NB)	GS (RRP)	pT	D’Amico	NCCN
miRNA-21	*rho* P-value	-0.065 0.677	0.107 0.496	0.071 0.653	0.104 0.508	0.169 0.279	0.133 0.397
miRNA-34a	*rho* P-value	0.124 0.425	-0.171 0.267	0.150 0.332	0.031 0.841	0.102 0.511	0.084 0.589
miRNA-125b	*rho* P-value	-0.145 0.355	-0.188 0.226	-0.135 0.390	-0.312 **0.042**	-0.149 0.339	-0.080 0.608
miRNA-126	*rho* P-value	-0.089 0.572	-0.019 0.905	0.088 0.574	-0.041 0.795	0.125 0.424	0.075 0.633
miRNA-143	*rho* P-value	0.023 0.883	-0.071 0.650	0.170 0.277	-0.004 0.982	0.013 0.935	-0.094 0.548
miRNA-145	*rho* P-value	-0.059 0.7038	0.204 0.1840	0.016 0.9197	-0.057 0.713	0.181 0.240	0.123 0.427

D’Amico: D’Amico Risk Profile; GS: Gleason score; NB: Needle biopsy; NCCN: National Comprehensive Cancer Network; P-values are from Spearman's rho test; PSA: Prostate specific antigen; pT: Pathological T-stage; RRP: Retropubic radical prostatectomy.

**Table 6:** Correlation between miRNAs expression in needle biopsies and relapse-free survival of 49 patients, who have been treated for local PCa with radical prostatectomy.

miRNA	HR	*p*-value	[95% CI]
miRNA-21	1.231	0.474	0.697 to 2.177
miRNA-34a	1.834	0.260	0.638 to 5.273
miRNA-125b	1.130	0.576	0.736 to 1.736
miRNA-126	1.083	0.742	0.674 to 1.740
miRNA-143	0.318	**0.016**	0.124 to 0.810
miRNA-145	0.910	0.778	0.473 to 1.750

CI: Confidence interval

HR: Hazard ratio

PCa: Prostate cancer

## Discussion

MiRNA expression profiling may differentiate between non-cancerous and cancerous prostate tissues and the role of miRNA as a potential clinical biomarker has been widely investigated. More than 50 miRNAs have been reported to be significantly expressed in PCa [34]. A plethora of studies on the role of miRNAs in the pathogenesis of PCa has increased our understanding of these promising biomarkers, however, also introduced a number of conflicting results (S1 Table). The stability of miRNA, even in FFPE tissue [35], allows studies on a large scale, but biological aspects such as tumor heterogeneity may have strong impact on the clinical and diagnostic utility of miRNA. Using TMAs containing benign and malignant prostate tissue cores in replicates, the main objective of our methodological study was to characterize and quantify miRNA expression to address tumor heterogeneity and its correlation to clinicopathological parameters.

For most of the studied miRNAs we found the ICC values of ISH expression signals among cores within patients to vary between 45% and 76% (Table 2). The intra-tumoral variation in miRNA expression may be caused by many factors, including difference between GS in the prostatectomy TMA cores. *Lu et al*. found that miRNA expression depends on tumor differentiation [17]. Consequently, heterogeneous miRNA expression is to be expected also in a PCa with several cancer foci varying in GS. *Walter et al*., using microarray profiling, have described similar heterogeneity of miRNA expression in two different tumor foci with different GS in the PCa specimen [36]. In addition, the amount of cancer cells and stroma in each TMA core from the same prostatectomy specimen varied widely, and therefore, the miRNA expression also reflected this compartmental variation in tissue composition.

From the clinical point of view, a robust biomarker should be associated with minimal “measurement noise” (= intra-patient variation), leaving the majority of the associated variation to the biological variation (= inter-patient variation). Thus, except for miRNA-125b and miRNA-21 showing intra-tumoral heterogeneity accounting for around 50% of the total variation, the investigated miRNAs showed ICC values from 63 to 76% and may represent potential biomarkers in PCa with more than 50% of the total variation attributable to inter-patient, biological variation. However, these overall findings do indicate a rather high intra-tumoral heterogeneity of miRNA-expression in this type of cancer.

Our study found miRNA-125b, miRNA-126, miRNA-143, and miRNA-145 to be represented primarily in the stromal compartment, while miRNA-21 and miRNA-34a were mainly expressed in epithelium (S3 Table). Focusing on individual miRNAs, our findings are corroborating previous observations by *Nonn et al*., who also reported higher miRNA-125b expression in the normal stroma compared to normal prostate epithelial cells [37]. *Walter et al*., using qPCR array profiling of manually microdissected tissue, found that miRNA-126 and miRNA-143 were tumor-associated stroma specific miRNAs, while miRNA-34a and miRNA-125b were represented in both epithelium and stroma in PCa [36]. Our findings are in contrast to those of *Peng et al*., who used LNA-based ISH and found lower or even absent expression of both miRNA-143 and miRNA-145 in tumor-associated stroma [38]. *Wach et al*. also noted that miRNA-143 resulted in a distinct cytoplasmic staining of malignant epithelial cells, whereas surrounding non-epithelial tissue did not show a staining signal [39]. MiRNA-143 and miRNA-145 are expressed from the same precursor transcript and would therefore be expected to partly correlate. Our ISH analysis identified both miRNAs in similar smooth muscle/fibroblastic stromal cells, which is a strong indication of specific ISH reactions and agrees with well-characterized expression of miRNA-143 and -145 in smooth muscle cells [40,41]. The discrepancy between our findings and those of Peng *et al*. is likely related to methodological differences as discussed in detail elsewhere [42].

We found a rather poor correlation between the miRNA expression in the pre-operative needle biopsy and the matched RRP specimen (Table 3), which is in contrast to the findings of *Nonn et al*. This conflicting result could be related to differences in the materials studied as well as the techniques used. In the study by *Nonn et al*., FFPE needle biopsies and frozen tissue from radical prostatectomies were compared. Moreover, the miRNA expression was assessed in normal prostate epithelium *versus* normal stroma and not in PCa (37). Furthermore, miRNA PCR array profiling was used instead of ISH. In addition, the differences in sample sizes may also explain some of the discrepancy. In our cohort, the amounts of both cancer epithelium and tumor-associated stroma were quite different in cancer cores of the TMA and the paired needle biopsies. Similarly, *Ren et al*. have demonstrated that the epithelial expressions of miRNA-30c, miRNA-27a, and miRNA-219 were quite different from the expression in stroma [43]. One should also consider the heterogeneity of miRNA expression in cancer epithelium compared to tumour-associated stroma [36,39], when evaluating these conflicting results.

Four of the investigated miRNAs, miRNA-21, miRNA-34a, miRNA-125b, and miRNA-126, showed upregulation in PCa cores of the TMA compared with their matched BPH cores suggesting a possible oncogenic role (Table 4). Regarding miRNA-21, this finding corroborates all previous studies. Interestingly, our ISH observations indicate that the increased levels measured in PCa are not related to expression in cancer cells, but rather to normal cells located in the hostile microenvironment (Fig 1). We found poor correlation of the miRNA-21 level of expression in PCa TMA cores and the matched BPH core, which suggests challenges as to clinical implementation of the miRNA-21 expression in needle biopsies. Our findings for the other three miRNAs are partially in conflict with previously reported studies of PCa (S1 Table). *Volinia et al*. found increased miRNA-34a levels in PCa using microarray [44], whereas other studies reported downregulation in PCa [45–47]. Using ISH in 24 PCa samples, *Lodygin et al*. found reduced levels of miRNA-34a in PCa compared to matched normal prostate tissue. However, when comparing with matched BPH, this reduction was less obvious [46]. For miRNA-125b we found a 2-fold increase in expression estimates comparing PCa and BPH. Inconsistent with this finding, several of the previous clinical studies have found downregulation of miRNA-125b in PCa compared to normal prostate tissue, except *Shi et al*., who found miRNA-125b overexpression in PCa by ISH [48]. Upregulation of miRNA-125b has also been reported in preclinical studies (cell lines PC3 [49] and LNCaP [50,51]). Interestingly, *Mitchell et al*. found increased levels of miRNA-125b in the PCa serum, when comparing samples from 25 metastatic prostate cancer patients with 25 age-matched male controls [52]. While miRNA-126 was overexpressed in PCa compared to non-cancerous prostate tissue in our study, all previous clinical studies have demonstrated downregulation in PCa. However, it is worth noting that these results have been obtained using either microarray or PCR, and not ISH. The technical platform and the type of material investigated may explain this discrepancy.

Although our main focus in this study was the heterogeneity of miRNA expression, we also looked for potential clinical relations. Only miRNA-143 showed marginal correlation to RFS (Table 6), its lower expression being associated with poor prognosis. This finding corresponds to the results obtained by *Clap et al*., who documented miRNA-143 to be considerably decreased in PCa by using ISH, and the expression was further decreased during progressive disease [53]. *Avgeris et al*. showed that downregulation of miRNA-145 expression in PCa predicted short disease free survival, independent of other prognostic factors such as GS, clinical stage of disease, and serum PSA levels [54]. This finding was consistent with the study of *Chen et al*., who also concluded that miRNA-145 expression may be a prognostic biomarker for the length of progression free survival [55]. These findings on miRNA-145 are not directly supported by our study.

Considering miRNA-143 and miRNA-145 as biological replicates, and since they originate from the same transcripts, the data taken together suggest that the miRNA-143 and miRNA-145 expressions are downregulated in PCa. Mature forms of miRNA-145 and miRNA-143 levels are regulated post-transcriptionally leading to differential levels in cells and tissue [56]. Differential processing during biogenesis and/or degradation of the two miRNAs are likely to explain such differences. In addition, the technical detection methods, like RT-qPCR, microarrays and NGS may have different specificities linked to the individual sequences of the two miRNAs. In the ISH analyses, the LNA probes are expected to detect both precursor and mature forms, which may help to explain differences in the results between the various platforms [57]. MiRNA-145 and miRNA-143 are involved in differentiation of smooth muscle through regulation of transcription factors like myocardin and Elk-1 [40]. Interestingly decreased levels of miRNA-143 may lead to increased TNF-a expression in smooth muscle cells [58]. Moreover smooth muscle is a prevalent stromal component of the prostate and reduced expression of miRNA-143 and miRNA-145 may be related to loss or de-differentiation of smooth muscle, possibly related to the aggressiveness of the cancer.

Except for the correlation between miRNA-125b expression and pT-stage, poor non-significant correlations were found between the studied miRNAs and the clinicopathological data of our cohort (Table 5). Previous studies have demonstrated a correlation between miRNA expression and pT, GS, PSA, and clinical stage of disease, although data are partly conflicting. For example, loss of miRNA-145 expression has been found inversely correlated with metastatic disease [38,59], GS, and PSA level [38,54,60]. Other studies are in accordance with our findings, *e*.*g*. *Wach et al*., who did not observe any correlation between miRNA-145 expression and GS, and including miRNA-143 did not reveal a correlation with pathologic stage of disease, either [39]. Moreover, *Kang et al*. did not find any significant correlation between miRNA-145 expression and the risk of BCR [61]. Limitations regarding assessment of the clinical impact of our data include the retrospective nature of our study, and the limited number of patients investigated.

## Conclusion

The present study documents intra-tumoral heterogeneity in the expression of various miRNAs, calling for caution in using these biomarkers in prognostic and predictive settings. The results, however, document upregulation of the expression of miRNA-21, miRNA-34a, miRNA-125, and miRNA-126 in PCa compared to BPH and may suggest a possible prognostic impact associated with the expression of miRNA-143.

## Supporting information

S1 Table Overview of some of these studies investigating the same miRNA panel used in the current study. (DOCX)

S2 Table Choice of signal intensity according to the individual microRNAs. (DOCX)

S3 Table Expressions of miRNAs in PCa in needle biopsy, in cancer cores and BPH of the TMAs. (DOCX)

S1 Dataset ICC of microRNAs expression in cancer cores in TMAs. (XLSX)

S2 Dataset microRNAs expression in cancer cores in TMAs vs in needle biopsy. (XLSX)

S3 Dataset microRNAs expression in cancer cores in vs in BPH in TMAs. (XLSX)

S4 Dataset microRNAs in needle biopsy and the clinico-pathological data. (XLSX)

S1 Fig Quantitation of miRNA by image analysis in prostate cancer. (DOCX)

## References

[1] Sundhedsdatastyrelsen. Nye Kræfttilfælde i Danmark. 2016;52.

[2] Sundhedsstyrelsen. Tal og analyse. Tal Og Anal. 2016;

[3] Ferlay J, Steliarova-Foucher E, Lortet-Tieulent J, Rosso S, Coebergh JWW, Comber H, et al Cancer incidence and mortality patterns in Europe: Estimates for 40 countries in 2012. Eur J Cancer [Internet]. 2013;49(6):1374–403. Available from: doi: 10.1016/j.ejca.2012.12.027 23485231 10.1016/j.ejca.2012.12.027

[4] Catalona WJ, Richie JP, Ahmann FR, Hudson MA, Scardino PT, Flanigan RC, et al Comparison of digital rectal examination and serum prostate specific antigen in the early detection of prostate cancer: results of a multicenter clinical trial of 6,630 men. J Urol [Internet]. 1994 5 [cited 2016 Jun 28];151(5):1283–90. Available from: http://www.ncbi.nlm.nih.gov/pubmed/7512659 7512659 10.1016/s0022-5347(17)35233-3

[5] Hoogendam A, Buntinx F, de Vet HC. The diagnostic value of digital rectal examination in primary care screening for prostate cancer: a meta-analysis. Fam Pract. 1999;16(6):621–6. 10625141 10.1093/fampra/16.6.621

[6] Daneshgari F, Taylor GD, Miller GJ, Crawford ED. Computer simulation of the probability of detecting low volume carcinoma of the prostate with six random systematic core biopsies. Urology. 1995;45(4):604–9. doi: 10.1016/S0090-4295(99)80051-X 7716840 10.1016/S0090-4295(99)80051-X

[7] Partin a W, Kattan MW, Subong EN, Walsh PC, Wojno KJ, Oesterling JE, et al Combination of prostate-specific antigen, clinical stage, and Gleason score to predict pathological stage of localized prostate cancer. A multi-institutional update. JAMA. 1997;277:1445–51. 9145716

[8] Gleason DF. Classification of prostatic carcinomas. Cancer Chemother Rep [Internet]. 1966 3 [cited 2015 Jan 21];50(3):125–8. Available from: http://www.ncbi.nlm.nih.gov/pubmed/5948714 5948714

[9] Boorjian SA, Karnes RJ, Crispen PL, Rangel LJ, Bergstralh EJ, Sebo TJ, et al The Impact of Discordance Between Biopsy and Pathological Gleason Scores on Survival After Radical Prostatectomy. J Urol. 2009;181(1):95–104. doi: 10.1016/j.juro.2008.09.016 19012937 10.1016/j.juro.2008.09.016

[10] Cohen MS, Hanley RS, Kurteva T, Ruthazer R, Silverman ML, Sorcini A, et al Comparing the Gleason Prostate Biopsy and Gleason Prostatectomy Grading System: The Lahey Clinic Medical Center Experience and an International Meta-Analysis. Eur Urol. 2008;54(2):371–81. doi: 10.1016/j.eururo.2008.03.049 18395322 10.1016/j.eururo.2008.03.049

[11] Villers A, McNeal JE, Freiha FS, Stamey TA. Multiple cancers in the prostate: Morphologic features of clinically recognized versus incidental tumors. Cancer. 1992;70(9):2313–8. 1382830 10.1002/1097-0142(19921101)70:9<2313::aid-cncr2820700917>3.0.co;2-t

[12] Wolters T, Montironi R, Mazzucchelli R, Scarpelli M, Roobol MJ, Van Den Bergh RCN, et al Comparison of incidentally detected prostate cancer with screen-detected prostate cancer treated by prostatectomy. Prostate. 2012;72(1):108–15. doi: 10.1002/pros.21415 21538424 10.1002/pros.21415

[13] Melia J, Moseley R, Ball RY, Griffiths DFR, Grigor K, Harnden P, et al A UK-based investigation of inter- and intra-observer reproducibility of Gleason grading of prostatic biopsies. Histopathology. 2006;48(6):644–54. doi: 10.1111/j.1365-2559.2006.02393.x 16681679 10.1111/j.1365-2559.2006.02393.x

[14] Mian BM, Lehr DJ, Moore CK, Fisher HAG, Kaufman RP, Ross JS, et al Role of prostate biopsy schemes in accurate prediction of Gleason scores. Urology [Internet]. 2006 2 [cited 2016 May 5];67(2):379–83. Available from: http://www.ncbi.nlm.nih.gov/pubmed/16461089 doi: 10.1016/j.urology.2005.08.018 16461089 10.1016/j.urology.2005.08.018

[15] Epstein Jonathan I MD; Egevad Lars MD, PhD; Amin Mahul B. MD; Delahunt Brett MD; Srigley John R. MD; Humphrey Peter A. MD P and the GC. The 2014 International Society of Urological Pathology (ISUP) Consensus Conference on Gleason Grading of Prostatic Carcinoma: Definition of Grading Patterns and Proposal for a New Grading System. Am J Surg Pathol [Internet]. 2016;40(2):244–52. Available from: http://www.ncbi.nlm.nih.gov/pubmed/26492179 10.1097/PAS.0000000000000530 26492179

[16] Danneman D, Drevin L, Delahunt B, Samaratunga H, Robinson D, Bratt O, et al The Accuracy of Prostate Biopsies for Predicting Gleason Score in Radical Prostatectomy Specimens. Nationwide trends 2000–2012. BJU Int [Internet]. 2016;1–7. Available from: http://www.ncbi.nlm.nih.gov/pubmed/26918298 10.1111/bju.13458 26918298

[17] Lu J, Getz G, Miska EA, Alvarez-Saavedra E, Lamb J, Peck D, et al MicroRNA expression profiles classify human cancers. Nature [Internet]. 2005;435(7043):834–8. Available from: http://www.ncbi.nlm.nih.gov/pubmed/15944708 doi: 10.1038/nature03702 15944708 10.1038/nature03702

[18] Carthew RW, Sontheimer EJ. Origins and Mechanisms of miRNAs and siRNAs. Cell [Internet]. Elsevier Inc.; 2009;136(4):642–55. Available from: doi: 10.1016/j.cell.2009.01.035 19239886 10.1016/j.cell.2009.01.035 PMC2675692

[19] Reinhart BJ, Weinstein EG, Rhoades MW, Bartel B, Bartel DP. MicroRNAs in plants. Genes Dev [Internet]. 2002 7 1 [cited 2016 Jun 9];16(13):1616–26. Available from: http://www.ncbi.nlm.nih.gov/pubmed/12101121 doi: 10.1101/gad.1004402 12101121 10.1101/gad.1004402 PMC186362

[20] Chen C-Z, Li L, Lodish HF, Bartel DP. MicroRNAs modulate hematopoietic lineage differentiation. Science [Internet]. 2004 1 2 [cited 2016 Jun 9];303(5654):83–6. Available from: http://www.ncbi.nlm.nih.gov/pubmed/14657504 doi: 10.1126/science.1091903 14657504 10.1126/science.1091903

[21] Bueno MJ, Pérez de Castro I, Malumbres M. Control of cell proliferation pathways by microRNAs. Cell Cycle [Internet]. 2008 10 [cited 2016 Jun 9];7(20):3143–8. Available from: http://www.ncbi.nlm.nih.gov/pubmed/18843198 doi: 10.4161/cc.7.20.6833 18843198 10.4161/cc.7.20.6833

[22] Xu P, Vernooy SY, Guo M, Hay BA. The Drosophila microRNA Mir-14 suppresses cell death and is required for normal fat metabolism. Curr Biol [Internet]. 2003 4 29 [cited 2016 Jun 9];13(9):790–5. Available from: http://www.ncbi.nlm.nih.gov/pubmed/12725740 12725740 10.1016/s0960-9822(03)00250-1

[23] Heneghan HM, Miller N, Kerin MJ. MiRNAs as biomarkers and therapeutic targets in cancer. Curr Opin Pharmacol [Internet]. Elsevier Ltd; 2010;10(5):543–50. Available from: doi: 10.1016/j.coph.2010.05.010 20541466 10.1016/j.coph.2010.05.010

[24] Bartels CL, Tsongalis GJ. MicroRNAs: novel biomarkers for human cancer. Clin Chem [Internet]. 2009 4 [cited 2016 Jun 9];55(4):623–31. Available from: http://www.ncbi.nlm.nih.gov/pubmed/19246618 doi: 10.1373/clinchem.2008.112805 19246618 10.1373/clinchem.2008.112805

[25] Kim WTW. MicroRNAs in prostate cancer. Prostate Int [Internet]. Published by Elsevier B.V on behalf of Prostate International; 2013;1(1):3–9. Available from: http://www.pubmedcentral.nih.gov/articlerender.fcgi?artid=3821523&tool=pmcentrez&rendertype=abstract doi: 10.12954/PI.12011 24223395 10.12954/PI.12011 PMC3821523

[26] Sobin LH, G.M. WC. TNM classification of malignant tumours—NLM Catalog—NCBI. UICC International Union Against Cancer 7th edn. 2009.

[27] Wilt Timothy J, Roderick MacDonald, Karen Hagerty, Paul Schellhammer, Kramer Barnett S. 5-Alpha-Reductase Inhibitors for Prostate Cancer Prevention. Cochrane Database Syst Rev [Internet]. 2008;(2):1–64. Available from: http://www.mrw.interscience.wiley.com/cochrane/clsysrev/articles/CD007091/frame.html 10.1002/14651858.CD007091 PMC11270836 18425978

[28] Mohler J, Bahnson RR, Boston B, Busby JE, Amico AD, Eastham J a, et al NCCN clinical practice guidelines in oncology: prostate cancer. Natl Compr Cancer Netw. 2010;8(2):162–200. 10.6004/jnccn.2010.0012 20141676

[29] D’Amico a V, Whittington R, Malkowicz SB, Schultz D, Blank K, Broderick G a, et al Biochemical outcome after radical prostatectomy, external beam radiation therapy, or interstitial radiation therapy for clinically localized prostate cancer. JAMA. 1998;280(11):969–74. 9749478 10.1001/jama.280.11.969

[30] Moul J. Prostate specific antigen only progression of prostate cancer. J Urol [Internet]. 2000;163(6):1632–42. Available from: http://www.ncbi.nlm.nih.gov/pubmed/10799151 10799151

[31] Epstein JI, Allsbrook WC, Amin MB, Egevad LL. The 2005 International Society of Urological Pathology (ISUP) Consensus Conference on Gleason Grading of Prostatic Carcinoma. Am J Surg Pathol [Internet]. 2005 9;29(9):1228–42. Available from: http://content.wkhealth.com/linkback/openurl?sid=WKPTLP:landingpage&an=00000478-200509000-00015 16096414 10.1097/01.pas.0000173646.99337.b1

[32] Nielsen BS, Jørgensen S, Fog JU, Søkilde R, Christensen IJ, Hansen U, et al High levels of microRNA-21 in the stroma of colorectal cancers predict short disease-free survival in stage II colon cancer patients. Clin Exp Metastasis [Internet]. 2011 1 [cited 2016 Aug 27];28(1):27–38. Available from: http://www.ncbi.nlm.nih.gov/pubmed/21069438 doi: 10.1007/s10585-010-9355-7 21069438 10.1007/s10585-010-9355-7 PMC2998639

[33] Mouritzen P, Nielsen AT, Pfundheller HM, Choleva Y, Kongsbak L, Møller S. Single nucleotide polymorphism genotyping using locked nucleic acid (LNA). Expert Rev Mol Diagn [Internet]. 2003 1 [cited 2016 May 7];3(1):27–38. Available from: http://www.ncbi.nlm.nih.gov/pubmed/12528362 doi: 10.1586/14737159.3.1.27 12528362 10.1586/14737159.3.1.27

[34] Cannistraci A, Di Pace AL, De Maria R, Bonci D. MicroRNA as new tools for prostate cancer risk assessment and therapeutic intervention: results from clinical data set and patients’ samples. Biomed Res Int [Internet]. 2014 1 [cited 2016 May 6];2014:146170 Available from: http://www.pubmedcentral.nih.gov/articlerender.fcgi?artid=4182080&tool=pmcentrez&rendertype=abstract doi: 10.1155/2014/146170 25309903 10.1155/2014/146170 PMC4182080

[35] Xi Y, Nakajima GO, Gavin E, Morris CG, Kudo K, Hayashi K, et al Systematic analysis of microRNA expression of RNA extracted from fresh frozen and formalin-fixed paraffin-embedded samples. Rna. 2007;13:1668–74. doi: 10.1261/rna.642907 17698639 10.1261/rna.642907 PMC1986820

[36] Walter BA, Valera VA, Pinto PA, Merino MJ. Comprehensive microRNA profiling of prostate cancer. J Cancer. 2013;4(5):350–7. doi: 10.7150/jca.6394 23781281 10.7150/jca.6394 PMC3677622

[37] Nonn L, Vaishnav A, Gallagher L, Gann PH. mRNA and micro-RNA expression analysis in laser-capture microdissected prostate biopsies: valuable tool for risk assessment and prevention trials. Exp Mol Pathol [Internet]. 2010 2 [cited 2016 May 5];88(1):45–51. Available from: http://www.pubmedcentral.nih.gov/articlerender.fcgi?artid=2815196&tool=pmcentrez&rendertype=abstract doi: 10.1016/j.yexmp.2009.10.005 19874819 10.1016/j.yexmp.2009.10.005 PMC2815196

[38] Peng X, Guo W, Liu T, Wang X, Tu X, Xiong D, et al Identification of miRs-143 and -145 that is associated with bone metastasis of prostate cancer and involved in the regulation of EMT. PLoS One. 2011;6(5). 10.1371/journal.pone.0020341 PMC3103579 21647377

[39] Wach S, Nolte E, Szczyrba J, Stöhr R, Hartmann A, Ørntoft T, et al MicroRNA profiles of prostate carcinoma detected by multiplatform microRNA screening. Int J Cancer. 2012;130(3):611–21. doi: 10.1002/ijc.26064 21400514 10.1002/ijc.26064

[40] Cordes KR, Sheehy NT, White MP, Berry EC, Morton SU, Muth AN, et al miR-145 and miR-143 regulate smooth muscle cell fate and plasticity. Nature [Internet]. Nature Publishing Group; 2009;460(7256):705–10. Available from: doi: 10.1038/nature08195 19578358 10.1038/nature08195 PMC2769203

[41] Chivukula RR, Shi G, Acharya A, Mills EW, Zeitels LR, Anandam JL, et al An essential mesenchymal function for miR-143/145 in intestinal epithelial regeneration. Cell [Internet]. Elsevier Inc.; 2014;157(5):1104–16. Available from: doi: 10.1016/j.cell.2014.03.055 24855947 10.1016/j.cell.2014.03.055 PMC4175516

[42] Nielsen BS. MicroRNA In Situ Hybridization. In: Methods in molecular biology (Clifton, NJ) [Internet]. 2012 [cited 2017 Jan 10]. p. 67–84. Available from: http://www.ncbi.nlm.nih.gov/pubmed/22144192 10.1007/978-1-61779-427-8_5 22144192

[43] Ren Q, Liang J, Wei J, Basturk O, Wang J, Daniels G, et al Epithelial and stromal expression of miRNAs during prostate cancer progression. Am J Transl Res. 2014;6(4):329–39. 25075250 PMC4113495

[44] Volinia S, Calin GA, Liu C-G, Ambs S, Cimmino A, Petrocca F, et al A microRNA expression signature of human solid tumors defines cancer gene targets. Proc Natl Acad Sci U S A [Internet]. 2006;103(7):2257–61. Available from: /pmc/articles/PMC1413718/?report=abstract doi: 10.1073/pnas.0510565103 16461460 10.1073/pnas.0510565103 PMC1413718

[45] Ambs S, Prueitt RL, Yi M, Hudson RS, Howe TM, Petrocca F, et al Genomic profiling of microRNA and messenger RNA reveals deregulated microRNA expression in prostate cancer. Cancer Res [Internet]. 2008 8 1 [cited 2013 Apr 17];68(15):6162–70. Available from: http://www.pubmedcentral.nih.gov/articlerender.fcgi?artid=2597340&tool=pmcentrez&rendertype=abstract doi: 10.1158/0008-5472.CAN-08-0144 18676839 10.1158/0008-5472.CAN-08-0144 PMC2597340

[46] Lodygin D, Tarasov V, Epanchintsev A, Berking C, Knyazeva T, Körner H, et al Inactivation of miR-34a by aberrant CpG methylation in multiple types of cancer. Cell Cycle. 2008;7(16):2591–600. doi: 10.4161/cc.7.16.6533 18719384 10.4161/cc.7.16.6533

[47] Yamamura S, Saini S, Majid S, Hirata H, Ueno K, Deng G, et al Microrna-34a modulates c-Myc transcriptional complexes to suppress malignancy in human prostate cancer cells. PLoS One. 2012;7(1). 10.1371/journal.pone.0029722 PMC3250472 22235332

[48] Shi XB, Xue L, Ma AH, Tepper CG, Kung HJ, White RWD. MiR-125b promotes growth of prostate cancer xenograft tumor through targeting pro-apoptotic genes. Prostate. 2011;71(5):538–49. doi: 10.1002/pros.21270 20886540 10.1002/pros.21270 PMC3017658

[49] Lee YS, Kim HK, Chung S, Kim KS, Dutta A. Depletion of human micro-RNA miR-125b reveals that it is critical for the proliferation of differentiated cells but not for the down-regulation of putative targets during differentiation. J Biol Chem. 2005;280(17):16635–41. doi: 10.1074/jbc.M412247200 15722555 10.1074/jbc.M412247200

[50] Shi X-B, Xue L, Yang J, Ma A-H, Zhao J, Xu M, et al An androgen-regulated miRNA suppresses Bak1 expression and induces androgen-independent growth of prostate cancer cells. Proc Natl Acad Sci U S A [Internet]. 2007 12 11;104(50):19983–8. Available from: http://www.pubmedcentral.nih.gov/articlerender.fcgi?artid=2148409&tool=pmcentrez&rendertype=abstract doi: 10.1073/pnas.0706641104 18056640 10.1073/pnas.0706641104 PMC2148409

[51] DeVere White RW, Vinall RL, Tepper CG, Shi X-B. MicroRNAs and their potential for translation in prostate cancer. Urol Oncol [Internet]. 1 [cited 2016 Jan 25];27(3):307–11. Available from: http://www.pubmedcentral.nih.gov/articlerender.fcgi?artid=2761743&tool=pmcentrez&rendertype=abstract doi: 10.1016/j.urolonc.2009.01.004 19414119 10.1016/j.urolonc.2009.01.004 PMC2761743

[52] Mitchell PS, Parkin RK, Kroh EM, Fritz BR, Wyman SK, Pogosova-Agadjanyan EL, et al Circulating microRNAs as stable blood-based markers for cancer detection. Proc Natl Acad Sci U S A [Internet]. 2008;105(30):10513–8. Available from: http://www.ncbi.nlm.nih.gov/pubmed/18663219 doi: 10.1073/pnas.0804549105 18663219 10.1073/pnas.0804549105 PMC2492472

[53] Clap?? C, Fritz V, Henriquet C, Apparailly F, Fernandez PL, Iborra F, et al miR-143 interferes with ERK5 signaling, and abrogates prostate cancer progression in mice. PLoS One. 2009;4(10):1–9. 10.1371/journal.pone.0007542 PMC2763222 19855844

[54] Avgeris M, Stravodimos EGF K and a S. The loss of the tumour-suppressor miR-145 results in the shorter disease-free survival of prostate cancer patients. Br J Cancer [Internet]. 2013;108(May):2573–81. Available from: http://dx.doi.org/10.1038/bjc.2013.250 23703249 10.1038/bjc.2013.250 PMC3694240

[55] Chen X, Gong J, Zeng H, Chen N, Huang R, Huang Y, et al MicroRNA145 targets BNIP3 and suppresses prostate cancer progression. Cancer Res. 2010;70(7):2728–38. doi: 10.1158/0008-5472.CAN-09-3718 20332243 10.1158/0008-5472.CAN-09-3718

[56] Hart M, Nolte E, Wach S, Szczyrba J, Taubert H, Rau TT, et al Comparative microRNA profiling of prostate carcinomas with increasing tumor stage by deep sequencing. Mol Cancer Res [Internet]. 2014;12(2):250–63. Available from: http://www.ncbi.nlm.nih.gov/pubmed/24337069 doi: 10.1158/1541-7786.MCR-13-0230 24337069 10.1158/1541-7786.MCR-13-0230

[57] Thorlacius-Ussing G, Schnack Nielsen B, Andersen V, Holmstrøm K, Pedersen AE. Expression and Localization of miR-21 and miR-126 in Mucosal Tissue from Patients with Inflammatory Bowel Disease. Inflamm Bowel Dis [Internet]. 2017;23(5):739–52. Available from: http://insights.ovid.com/crossref?an=00054725-201705000-00009 doi: 10.1097/MIB.0000000000001086 28426456 10.1097/MIB.0000000000001086

[58] Lakhkar A, Dhagia V, Joshi SR, Gotlinger K, Patel D, Sun D, et al 20-HETE-induced mitochondrial superoxide production and inflammatory phenotype in vascular smooth muscle is prevented by glucose-6-phosphate dehydrogenase inhibition. Am J Physiol Heart Circ Physiol. 2016;310(9):H1107–17. doi: 10.1152/ajpheart.00961.2015 26921441 10.1152/ajpheart.00961.2015 PMC4867393

[59] Watahiki A, Wang Y, Morris J, Dennis K, O’Dwyer HM, Gleave M, et al MicroRNAs associated with metastatic prostate cancer. PLoS One [Internet]. 2011 1 [cited 2013 Mar 4];6(9):e24950 Available from: http://www.pubmedcentral.nih.gov/articlerender.fcgi?artid=3184096&tool=pmcentrez&rendertype=abstract doi: 10.1371/journal.pone.0024950 21980368 10.1371/journal.pone.0024950 PMC3184096

[60] Wang L, Tang H, Thayanithy V, Subramanian S, Oberg AL, Cunningham JM, et al Gene networks and microRNAs implicated in aggressive prostate cancer. Cancer Res [Internet]. 2009 12 15 [cited 2013 Apr 3];69(24):9490–7. Available from: http://www.pubmedcentral.nih.gov/articlerender.fcgi?artid=2795036&tool=pmcentrez&rendertype=abstract doi: 10.1158/0008-5472.CAN-09-2183 19996289 10.1158/0008-5472.CAN-09-2183 PMC2795036

[61] Kang SG, Ha YR, Kim SJ, Kang SH, Park HS, Lee JG, et al Do microRNA 96, 145 and 221 expressions really aid in the prognosis of prostate carcinoma? Asian J Androl [Internet]. 2012;14(5):752–7. Available from: http://www.pubmedcentral.nih.gov/articlerender.fcgi?artid=3734986&tool=pmcentrez&rendertype=abstract doi: 10.1038/aja.2012.68 22864280 10.1038/aja.2012.68 PMC3734986

