# Supplementary material for: Heterogeneity of miRNA expression in localized prostate cancer with clinicopathological correlations
Source: PLoS One. 2017 Jun 19;12(6):e0179113. doi: 10.1371/journal.pone.0179113 (PMC5476257; doi:10.1371/journal.pone.0179113)
Supplement: S1 Table — (DOCX) [file pone.0179113.s001.docx]

| Author | year | Material | No# | Method | Results |
| --- | --- | --- | --- | --- | --- |
| miR-21 | | | | | |
| Volinia et al[1] | 2006 | NA | 56 PCa 7 non-cancerous prostate tissue. | Microarray | Upregulated in PCa vs. non malignant tissue |
| Wang et al[2] | 2008 | FF | 10 androgen-independent PCa. 10 androgen dependent PCa. | Microarray | miRNA-21 demonstrates differential expression in PCa, comparing with normal tissues |
| Ribas et al[3] | 2009 | FF | 10 matched PCa. ttt by RP with normal tissue | Microarray | miRNA-21 is upregulated in PCa compared to normal prostate tissue. |
| Szczyrba et al[4] | 2010 | FF | 10 unmatched PCa ttt. by RP, and normal prostate tissue.  26 matched PCa and non-cancerous tissue | Northern blotting  Quantitative real-time PCR | miRNA-21 is upregulated in PCa. |
| Leite et al[5] | 2011 | FF | 49 PCa ttt by RP 10 BPH | Quantitative reverse transcriptase PCR | miRNA-21 didn't show significant correlation to biochemical recurrence. |
| Jalava et al[6] | 2012 | FF | 28 PCa 14 CRPCa 12 BPH | Microarray | miRNA-21 was significantly overexpressed in CRPCa. |
| Li et al[7] | 2012 | FFPE | 169 PCa ttt. by RP. | ISH | Positive miRNA-21 expression was associated with poor biochemical recurrence-free survival and predicted the risk of biochemical recurrence. |
| Amankwah et al[8] | 2013 | FFPE | 28 recurrent PCa 37 non-recurrent Pca (Both ttt. by RP) | Quantitative real-time PCR | Low miRNA-21 is associated with BCR. Differential expression of miR-21 is more prominent in obese than in non-obese cases. |
| Melbø-Jørgensen et al[9] | 2014 | FFPE | 535 PCa ttt by RP | Quantitative real-time PCR  ISH | High stromal expression of miR-21 was associated with poor biochemical recurrence-free survival after RP |
| Ren et al[10] | 2014 | FFPE | Set I: 204 PCa and 11 BPH  Set II: 124 PCa and 22 BPH | Microarray  ISH | Downregulation of miRNA-21 is associated with metastatic disease |
| miR-34a | | | | | |
| Volinia et al[1] | 2006 | NA | 56 PCa 7 non-cancerous prostate tissue. | Microarray | Upregulated in PCa vs. non-malignant PCa tissue |
| Ambs et al[11] | 2008 | FF | 60 PCa ttt. by RP 16 non-cancerous prostate tissue | Microarray Quantitative real-time PCR | Downregulation of 34a in PCa tissue. |
| Lodygin et al[12] | 2008 | FFPE | 24 PCa ttt. by RP with their matched normal and BPH tissue. | Real-time PCR ISH | Downregulation of 34a in PCa tissue. |
| Kong et al[13] | 2012 | FFPE | 127 PCa and matched adjacent normal tissues ttt. By RP. 11 patients who underwent experimental intervention and then ttt. by RP | Real-time PCR | Loss of miR-34a is consistent with increased expression of AR. |
| Yamamura et[14] al | 2012 | FFPE | 10 PCa and matched adjacent normal tissues. | Quantitative real-time PCR | miR-34a expression is downregulated in PCa. |
| miR-125b | | | | | |
| Porkka et al[15] | 2007 | FF | 9 PCa ( 4 untreated PCa and 5 CRPCa) 4 BPH | Microarrays Quantitative real-time PCR | Downregulation of miRNA-125b in all PCa |
| Shi et al[16] | 2007 | FFPE | 10 PCa and 2 BPH | ISH | Upregulated in PCa vs. BPH.  Overexpression of miRNA-125b is correlated with GS. |
| Ozen et al[17] | 2008 | FF | 16 PCa ttt. by RP, and 30 PCa for validation. 10 benign peripheral zone tissues | Microarrays. Real-time PCR | Downregulation of miRNA-125b in PCa |
| Tong et al[18] | 2009 | FFPE | 40 PCa ttt. by RP. | Microarray Quantitative reverse transcriptase PCR | miRNA-125b was reduced in PCa. |
| Spahn et al[19] | 2010 | FFPE | 12 PCa ttt. RP 12 matched LN mets. 9 non neoplastic. | Microarray Quantitative real-time PCR | Downregulation of miRNA-125b in PCa tissues and matched LN mets. |
| Szczyrba et al[4] | 2010 | FF | 10 unmatched PCa ttt. by RP, and normal prostate tissue.  26 matched PCa and non-cancerous tissue | Northern blotting  Quantitative real-time PCR | miRNA-125b is downregulated in PCa. |
| Schaefer et al[20] | 2010 | FF | 76 PCa and matched adjacent normal tissue ttt. by RP. | Microarray Quantitative real-time PCR | Downregulation of miRNA-125b in PCa  miRNA-125 expression correlates with tumor stage. |
| Peng et al[21] | 2011 | FFPE | 6 primary and 7 bone metastatic PCa samples 16 primary PCa and 13 bone metastases samples | Microarray Quantitative reverse transcriptase PCR  ISH | Downregulation of miRNA-125b in mPCa compared to primary PCa. |
| Walter et al[22] | 2013 | NA | 37 PCa and matched adjcaent normal tissue ttt. by RP. | Real-time PCR | Downregulation of miRNA-125b in PCa |
| miR-126 | | | | | |
| Ambs et al[11] | 2008 | FF | 60 PCa ttt. by RP 16 non-cancerous prostate tissue | Microarray. Quantitative real-time PCR | Downregulation of miRNA-126 in PCa |
| Saito et al[23] | 2008 | NA | 4 PCa matched with adjacent normal tissue. | Real-time PCR. | miRNA-126 is downregulated in primary tumors. |
| Prueitt et al[24] | 2008 | FF | 57 PCa ttt. by RP (50 with PNI and 7 without) | Microarray. Quantitative real-time PCR | miRNA-126 is upregulated in tumors with PNI |
| Walter et al[22] | 2013 | NA | 37 PCa and matched adjcaent normal tissue ttt. by RP. | Real-time PCR | Downregulation of miRNA-126 with increasing GS |
| miR-143 | | | | | |
| Porkka et al[15] | 2007 | FF | 9 PCa ( 4 untretaed PCa and 5 CRPCa) 4 BPH | Microarrays Quantitative real-time PCR | Downregulation of miRNA-143 in all carcinomas |
| Tong et al[18] | 2009 | FFPE | 40 PCa ttt. by RP. | Microarray Quantitative reverse transcriptase PCR | miRNA-143 was significantly reduced in PCa |
| Clap et al[25] | 2009 | FFPE | 37 unmatched samples (13 normal tissue and 24 PCa) One matched PCa and adjacent normal tissue. | Quantitative real-time PCR ISH | miRNA-143 levels are inversely correlated with advanced stages of PCa. |
| Szczyrba et al[4] | 2010 | FF | 10 unmatched PCa ttt. by RP, and normal prostate tissue.  26 matched PCa and non-cancerous tissue | Northern blotting  Quantitative real-time PCR | miRNA-143 is downregulated in PCa. |
| Peng et al[21] | 2011 | FFPE | 6 primary and 7 bone metastatic PCa samples 16 primary PCa and 13 bone metastases samples | Microarray Quantitative reverse transcriptase PCR  ISH | The expressions of miRNA-143was downregulated significantly in metastasis samples. Downregulation of miRNA-143 was negatively correlated to bone metastasis, the Gleason score and level of free PSA in primary PCa. |
| Martens-Uzunova et al [26] | 2012 | FF | 102 matched PCa ttt. by RP and normal tissue. | Illumina sequencing Microarray Quantitative reverse transcriptase PCR | Downregulation of miRNA-143 |
| Wach et al[27] | 2012 | Set I: FFPE Set II: FF | Set I: 50 matched PCa ttt. by RP and adjacent normal tissue. Set II:26 matched PCa ttt. by RP and adjacent normal tissue. | Microarrays. Quantitative real-time PCR. ISH | Lower expression of miRNA-143 correlated to less differentiated tumor, poorly differentiated tumors expressed a reduced amount of miR-143 |
| Carlsson et al[28] | 2013 | FFPE | 13 PCa ttt. by RP (5 from TZ, 5 from PZ and 3 from both)  10 normal prostate tissue (one sample from TZ and one from PZ) | Microarray | miRNA-143 expression differentiated normal TZ from malignant TZ with 94% accuracy. |
| Hart et al[29] | 2014 | Set 1: FFPE  Set 2: FF | 20 matched PCa and their adjacent normal prostate tissue.  40 matched PCa and their matched normal prostate tissue. | Illumina sequencing Quantitative real-time PCR. | Downregulation of miRNA-143 in PCa compared to adjacent non-neoplastic tissue. |
| miR-145 | | | | | |
| Porkka et al[15] | 2007 | FF | 9 PCa samples ( 4 untretaed PCa and 5 CRPCa) 4 BPH | Microarrays Quantitative real-time PCR | Downregulation of 145 in all carcinomas |
| Ozen et al[17] | 2008 | FF | 16 PCa ttt. by RP, and 30 PCa for validation. 10 benign peripheral zone tissues | Microarrays. Real-time PCR | Downregulation of 145 in PCa tissues |
| Prueitt et al[24] | 2008 | FF | 57 PCa ttt. by RP (50 with PNI and 7 without) | Microarray. Quantitative real-time PCR | Downregulation of miRNA-145 |
| Ambs et al[11] | 2008 | FF | 60 PCa ttt. by RP 16 non-cancerous prostate tissue | Microarray. Quantitative real-time PCR | Downregulation of miRNA-145 in PCa tissue. |
| Tong et al[18] | 2009 | FFPE | 40 PCa ttt. by RP. | Microarray Quantitative reverse transcriptase PCR | miRNA-145 was significantly reduced in malignant prostate cancers. |
| Chen et al[30] | 2010 | FFPE and FF | 217 samples, including 134 PCa (121 needle biopsies, 13 TUR-P) and 83 benign prostate TUR-P snap-frozen fresh tissue samples (2 PCa, 3 BPH, and 2 normal). | Quantitative real-time PCR  Quantitative reverse transcriptase PCR ISH. | miRNA-145 was significantly downregulated in prostate cancer. |
| Zaman et al[31] | 2010 | FFPE | 27 matched PCa ttt. by RP and adjacent normal tissue. | Quantitative real-time PCR | Downregulated miRNA-145. |
| Schaefer et al[20] | 2010 | FF | 76 PCa and matched adjacent normal tissue ttt. by RP. | Microarray Quantitative real-time PCR | Downregulation of miRNA-145 in PCa tissues |
| Szczyrba et al[4] | 2010 | FF | 10 unmatched PCa ttt. by RP, and normal prostate tissue.  26 matched PCa and non-cancerous tissue | Northern blotting  Quantitative real-time PCR | miRNA-145 is downregulated in PCa. |
| Peng et al[21] | 2011 | FFPE | 6 primary and 7 bone metastatic PCa samples 16 primary PCa and 13 bone metastases samples | Microarray Quantitative reverse transcriptase PCR  ISH | The expressions of miRs-143 and -145 were down-regulate significantly in metastasis samples. Down-regulations of miRs-143 and -145 were negatively correlated to bone metastasis, the Gleason score and level of free PSA in primary PCa. |
| Wach et al[27] | 2012 | Set I: FFPE Set II: FF | Set I: 50 matched PCa ttt. by RP and adjacent normal tissue. Set II: 26 matched PCa ttt. by RP and adjacent normal tissue. | Microarrays. Quantitative real-time PCR. ISH | miRNA-145 has no correlation with Gleason sum or Gleason components. |
| Martens-Uzunova et al [26] | 2012 | FF | 102 matched PCa ttt. by RP and normal tissue. | Illumina sequencing Microarray Quantitative reverse transcriptase PCR | Downregulation of miRNA-145 |
| Fuse et al[32] | 2012 | NA | 20 PCa ttt by RP.  22 non-cancerous PCa | Microarrays. Quantitative real-time PCR. | miRNA-145 is downregulated in PCa. |
| Kang et al[33] | 2012 | FFPE | 73 PCa ttt. by RP. | Quantitative reverse transcriptase PCR | No significant correlation between the expression of miRNA-145 and clinicopathologic parameters |
| Avgeris et al[34] | 2013 | FF | 73 PCa ttt. by RP. 64 BPH | Quantitative real-time PCR. | Downregulated miRNA-145 expression in PCa compared with BPH patients. The reduction of miRNA-145 expression in PCa was correlated with higher GS, advanced clinical stage, larger tumour diameter and higher PSA and follow-up PSA levels. Higher risk for BCR and shorter DFS was found for the PCa patients expressing lower miRNA-145. |
| Hart et al[35] | 2013 | FF | 26 matched PCa and their adjacent normal tissue. | ISH Quantitative real-time PCR. | Downregulation of miRNA-145 in PCa compared to adjacent non-neoplastic tissue. |
| Hart et al[29] | 2014 | Set 1: FFPE  Set 2: FF | 20 matched PCa and their adjacent normal prostate tissue.  40 matched PCa and their matched normal prostate tissue. | Illumina sequencing Quantitative real-time PCR. | Downregulation of miRNA-145 in PCa compared to adjacent non-neoplastic tissue. |

**Supplementary S1 Table: Overview of some of these studies investigating the same miRNA panel used in the current study.**

AR: Androgen receptor; BCR: biochemical recurrence; BPH: Benign prostate hyperplasia; CRPCa: Castration resistant prostate cancer; DFS: disease-free survival; FFPE: Formalin fixed paraffin embedded samples; FF: Freshly frozen samples; GS: Gleason score; ISH: In situ hybridization; LN mets: Lymph nodes metastases; NA: Not available;, PCa: prostate cancer; PNI: perineural invasion; PSA: prostate-specific antigen; RP: Radical prostatectomy; TUR-P: transurethral resection of prostate.

**References**

1. Volinia S, Calin GA, Liu C-G, Ambs S, Cimmino A, Petrocca F, et al. A microRNA expression signature of human solid tumors defines cancer gene targets. Proc Natl Acad Sci U S A [Internet]. 2006;103(7):2257–61. Available from: /pmc/articles/PMC1413718/?report=abstract

2. Wang G, Wang Y, Feng W, Wang X, Yang JY, Zhao Y, et al. Transcription factor and microRNA regulation in androgen-dependent and -independent prostate cancer cells. BMC Genomics [Internet]. 2008;9 Suppl 2:S22. Available from: http://www.pubmedcentral.nih.gov/articlerender.fcgi?artid=2559887&tool=pmcentrez&rendertype=abstract

3. Ribas J, Ni X, Haffner M, Wentzel EA, Salmasi AH, Chowdhury WH, et al. miR-21: An androgen receptor-regulated microRNA that promotes hormone-dependent and hormone-independent prostate cancer growth. Cancer Res. 2009;69(18):7165–9.

4. Szczyrba J, Löprich E, Wach S, Jung V, Unteregger G, Barth S, et al. The microRNA profile of prostate carcinoma obtained by deep sequencing. Mol Cancer Res [Internet]. 2010 Apr [cited 2013 Apr 9];8(4):529–38. Available from: http://www.ncbi.nlm.nih.gov/pubmed/20353999

5. Leite KRM, Tomiyama A, Reis ST, Sousa-Canavez JM, Saudo A, Dall’Oglio MF, et al. MicroRNA-100 expression is independently related to biochemical recurrence of prostate cancer. J Urol [Internet]. American Urological Association Education and Research, Inc.; 2011;185(3):1118–22. Available from: http://dx.doi.org/10.1016/j.juro.2010.10.035

6. Jalava SE, Urbanucci a, Latonen L, Waltering KK, Sahu B, Jänne O a, et al. Androgen-regulated miR-32 targets BTG2 and is overexpressed in castration-resistant prostate cancer. Oncogene. 2012;31(41):4460–71.

7. Li T, Li RS, Li YH, Zhong S, Chen YY, Zhang CM, et al. MiR-21 as an independent biochemical recurrence predictor and potential therapeutic target for prostate cancer. J Urol. 2012;187(4):1466–72.

8. Amankwah EK, Anegbe E, Park H, Pow-Sang J, Hakam A, Park JY. miR-21, miR-221 and miR-222 expression and prostate cancer recurrence among obese and non-obese cases. Asian J Androl [Internet]. 2013;15(2):226–30. Available from: http://www.pubmedcentral.nih.gov/articlerender.fcgi?artid=3705740&tool=pmcentrez&rendertype=abstract

9. Melbø-Jørgensen C, Ness N, Andersen S, Valkov A, Dønnem T, Al-Saad S, et al. Stromal expression of MiR-21 predicts biochemical failure in prostate cancer patients with Gleason score 6. Aoki I, editor. PLoS One [Internet]. 2014 Nov 17 [cited 2017 Mar 9];9(11):e113039. Available from: http://dx.plos.org/10.1371/journal.pone.0113039

10. Ren Q, Liang J, Wei J, Basturk O, Wang J, Daniels G, et al. Epithelial and stromal expression of miRNAs during prostate cancer progression. Am J Transl Res. 2014;6(4):329–39.

11. Ambs S, Prueitt RL, Yi M, Hudson RS, Howe TM, Petrocca F, et al. Genomic profiling of microRNA and messenger RNA reveals deregulated microRNA expression in prostate cancer. Cancer Res [Internet]. 2008 Aug 1 [cited 2013 Apr 17];68(15):6162–70. Available from: http://www.pubmedcentral.nih.gov/articlerender.fcgi?artid=2597340&tool=pmcentrez&rendertype=abstract

12. Lodygin D, Tarasov V, Epanchintsev A, Berking C, Knyazeva T, Körner H, et al. Inactivation of miR-34a by aberrant CpG methylation in multiple types of cancer. Cell Cycle. 2008;7(16):2591–600.

13. Kong D, Heath E, Chen W, Cher M, Powell I, Heilbrun L, et al. Epigenetic silencing of miR-34a in human prostate cancer cells and tumor tissue specimens can be reversed by BR-DIM treatment. Am J Transl Res [Internet]. 2012;4(1):14–23. Available from: http://www.embase.com/search/results?subaction=viewrecord&from=export&id=L364196755%5Cnhttp://www.ajtr.org/files/AJTR1111004.pdf%5Cnhttp://limo.libis.be/resolver?&sid=EMBASE&issn=19438141&id=doi:&atitle=Epigenetic+silencing+of+miR-34a+in+human+prostate+cancer

14. Yamamura S, Saini S, Majid S, Hirata H, Ueno K, Deng G, et al. Microrna-34a modulates c-Myc transcriptional complexes to suppress malignancy in human prostate cancer cells. PLoS One. 2012;7(1).

15. Porkka KP, Pfeiffer MJ, Waltering KK, Vessella RL, Tammela TLJ, Visakorpi T. MicroRNA expression profiling in prostate cancer. Cancer Res [Internet]. 2007 Jul 1 [cited 2013 Feb 28];67(13):6130–5. Available from: http://www.ncbi.nlm.nih.gov/pubmed/17616669

16. Shi X-B, Xue L, Yang J, Ma A-H, Zhao J, Xu M, et al. An androgen-regulated miRNA suppresses Bak1 expression and induces androgen-independent growth of prostate cancer cells. Proc Natl Acad Sci U S A [Internet]. 2007 Dec 11;104(50):19983–8. Available from: http://www.pubmedcentral.nih.gov/articlerender.fcgi?artid=2148409&tool=pmcentrez&rendertype=abstract

17. Ozen M, Creighton CJ, Ozdemir M, Ittmann M. Widespread deregulation of microRNA expression in human prostate cancer. Oncogene. 2008;27(12):1788–93.

18. Tong a W, Fulgham P, Jay C, Chen P, Khalil I, Liu S, et al. MicroRNA profile analysis of human prostate cancers. Cancer Gene Ther [Internet]. 2009 Mar [cited 2013 Apr 17];16(3):206–16. Available from: http://www.ncbi.nlm.nih.gov/pubmed/18949015

19. Spahn M, Kneitz S, Scholz CJ, Stenger N, Rüdiger T, Ströbel P, et al. Expression of microRNA-221 is progressively reduced in aggressive prostate cancer and metastasis and predicts clinical recurrence. Int J Cancer. 2010;127(2):394–403.

20. Schaefer A, Jung M, Mollenkopf H-J, Wagner I, Stephan C, Jentzmik F, et al. Diagnostic and prognostic implications of microRNA profiling in prostate carcinoma. Int J Cancer [Internet]. 2010;126(5):1166–76. Available from: http://www.hubmed.org/display.cgi?uids=19676045

21. Peng X, Guo W, Liu T, Wang X, Tu X, Xiong D, et al. Identification of miRs-143 and -145 that is associated with bone metastasis of prostate cancer and involved in the regulation of EMT. PLoS One. 2011;6(5).

22. Walter BA, Valera VA, Pinto PA, Merino MJ. Comprehensive microRNA profiling of prostate cancer. J Cancer. 2013;4(5):350–7.

23. Saito Y, Friedman JM, Chihara Y, Egger G, Chuang JC, Liang G. Epigenetic therapy upregulates the tumor suppressor microRNA-126 and its host gene EGFL7 in human cancer cells. Biochem Biophys Res Commun [Internet]. Elsevier Inc.; 2009;379(3):726–31. Available from: http://dx.doi.org/10.1016/j.bbrc.2008.12.098

24. Prueitt RL, Yi M, Hudson RS, Wallace TA, Howe TM, Yfantis HG, et al. Expression of microRNAs and protein-coding genes associated with perineural invasion in prostate cancer. Prostate. 2008;68(11):1152–64.

25. Clapé C, Fritz V, Henriquet C, Apparailly F, Fernandez PL, Iborra F, et al. miR-143 interferes with ERK5 signaling, and abrogates prostate cancer progression in mice. Creighton C, editor. PLoS One [Internet]. 2009 Oct 26 [cited 2017 Mar 9];4(10):e7542. Available from: http://dx.plos.org/10.1371/journal.pone.0007542

26. Martens-Uzunova ES, Jalava SE, Dits NF, van Leenders GJLH, Møller S, Trapman J, et al. Diagnostic and prognostic signatures from the small non-coding RNA transcriptome in prostate cancer. Oncogene. 2012;31(8):978–91.

27. Wach S, Nolte E, Szczyrba J, Stöhr R, Hartmann A, Ørntoft T, et al. MicroRNA profiles of prostate carcinoma detected by multiplatform microRNA screening. Int J Cancer. 2012;130(3):611–21.

28. Carlsson J, Helenius G, Karlsson MG, Andrén O, Klinga-Levan K, Olsson B. Differences in microRNA expression during tumor development in the transition and peripheral zones of the prostate. BMC Cancer [Internet]. 2013;13:362. Available from: http://www.pubmedcentral.nih.gov/articlerender.fcgi?artid=3733730&tool=pmcentrez&rendertype=abstract

29. Hart M, Nolte E, Wach S, Szczyrba J, Taubert H, Rau TT, et al. Comparative microRNA profiling of prostate carcinomas with increasing tumor stage by deep sequencing. Mol Cancer Res [Internet]. 2014;12(2):250–63. Available from: http://www.ncbi.nlm.nih.gov/pubmed/24337069

30. Chen X, Gong J, Zeng H, Chen N, Huang R, Huang Y, et al. MicroRNA145 targets BNIP3 and suppresses prostate cancer progression. Cancer Res. 2010;70(7):2728–38.

31. Zaman MS, Chen Y, Deng G, Shahryari V, Suh SO, Saini S, et al. The functional significance of microRNA-145 in prostate cancer. Br J Cancer [Internet]. 2010;103(2):256–64. Available from: http://www.pubmedcentral.nih.gov/articlerender.fcgi?artid=2906737&tool=pmcentrez&rendertype=abstract

32. Fuse M, Kojima S, Enokida H, Chiyomaru T, Yoshino H, Nohata N, et al. Tumor suppressive microRNAs (miR-222 and miR-31) regulate molecular pathways based on microRNA expression signature in prostate cancer. J Hum Genet [Internet]. Nature Publishing Group; 2012;57(11):691–9. Available from: http://www.ncbi.nlm.nih.gov/pubmed/22854542

33. Kang SG, Ha YR, Kim SJ, Kang SH, Park HS, Lee JG, et al. Do microRNA 96, 145 and 221 expressions really aid in the prognosis of prostate carcinoma? Asian J Androl [Internet]. 2012;14(5):752–7. Available from: http://www.pubmedcentral.nih.gov/articlerender.fcgi?artid=3734986&tool=pmcentrez&rendertype=abstract

34. M Avgeris, K Stravodimos EGF and a S. The loss of the tumour-suppressor miR-145 results in the shorter disease-free survival of prostate cancer patients. Br J Cancer [Internet]. 2013;108(May):2573–81. Available from: http://dx.doi.org/10.1038/bjc.2013.250

35. Hart M, Wach S, Nolte E, Szczyrba J, Menon R, Taubert H, et al. The proto-oncogene ERG is a target of microRNA miR-145 in prostate cancer. FEBS J. 2013;280(9):2105–16.
