# Supplementary material for: Heterogeneity of miRNA expression in localized prostate cancer with clinicopathological correlations
Source: PLoS One. 2017 Jun 19;12(6):e0179113. doi: 10.1371/journal.pone.0179113 (PMC5476257; doi:10.1371/journal.pone.0179113)
Supplement: S2 Table — (DOCX) [file pone.0179113.s002.docx]

**Supplementary S2 Table: Choice of signal intensity according to the individual microRNAs**

| miRNA | Signal | Reason |
| --- | --- | --- |
| miRNA-21 | Weak and intense | Both signals are weakly represented in region of interest |
| miRNA-34a | Weak and intense | Both signals are weakly represented in region of interest |
| miRNA-125b | Intense | Weak signal is almost represented only in stroma |
| miRNA-126 | Intense | Weak signal is almost represented only in stroma |
| miRNA-143 | Weak | Intense signal is represented almost only in uninterested tissue (blood vessels and fibromuscular stroma) |
| miRNA-145 | Weak and intense | Both signals are weakly represented in region of interest |
