# Supplementary material for: Heterogeneity of miRNA expression in localized prostate cancer with clinicopathological correlations
Source: PLoS One. 2017 Jun 19;12(6):e0179113. doi: 10.1371/journal.pone.0179113 (PMC5476257; doi:10.1371/journal.pone.0179113)
Supplement: S3 Table — (DOCX) [file pone.0179113.s003.docx]

| miRNA | Cancer in biopsy | Cancer cores in TMA | BPH |
| --- | --- | --- | --- |
| miRNA-21 | Weak and most predominant in normal-benign glandular epithelium. | Weak expression in both stromal and epithelial structures | Absent in the tissue |
| miRNA-34a | Intense signal most often seen in the cancer epithelial compartment. | Absent in stroma | Prevalent in the epithelium |
| miRNA-125b | Generally seen in fibroblastic stromal cells, but variation was identified | Prevalent in the stroma | Weak in the stroma |
| miRNA-126 | Generally seen in endothelial cells | Seen in endothelial cells vessels | Seen in endothelial cells vessels |
| miRNA-143 | Predominant expressed in the stroma and the vascular smooth muscle had the most intense signal. The stroma was often stained in the whole area, not limited to staining around the nuclei. | Primarily cytoplasmic expression in fibroblastic cells | Cytoplasmic in fibroblastic cells. |
| miRNA-145 | As miRNA-143 but less intense and more defined | Expressed in cytoplasm and intense in the nuclei of fibroblastic cells | Weak expression in the cytoplasm and intense in the nuclei of fibroblastic cells |

**Supplementary S3 Table: Expressions of miRNAs in PCa in needle biopsy, in cancer cores and BPH of the TMA**

BPH: Benign prostate hyperplasia

PCa: Prostate cancer

TMA: Tissue microarray
