# Supplementary material for: Heterogeneity of miRNA expression in localized prostate cancer with clinicopathological correlations
Source: PLoS One. 2017 Jun 19;12(6):e0179113. doi: 10.1371/journal.pone.0179113 (PMC5476257; doi:10.1371/journal.pone.0179113)
Supplement: S1 Fig — (DOCX) [file pone.0179113.s008.docx]

**
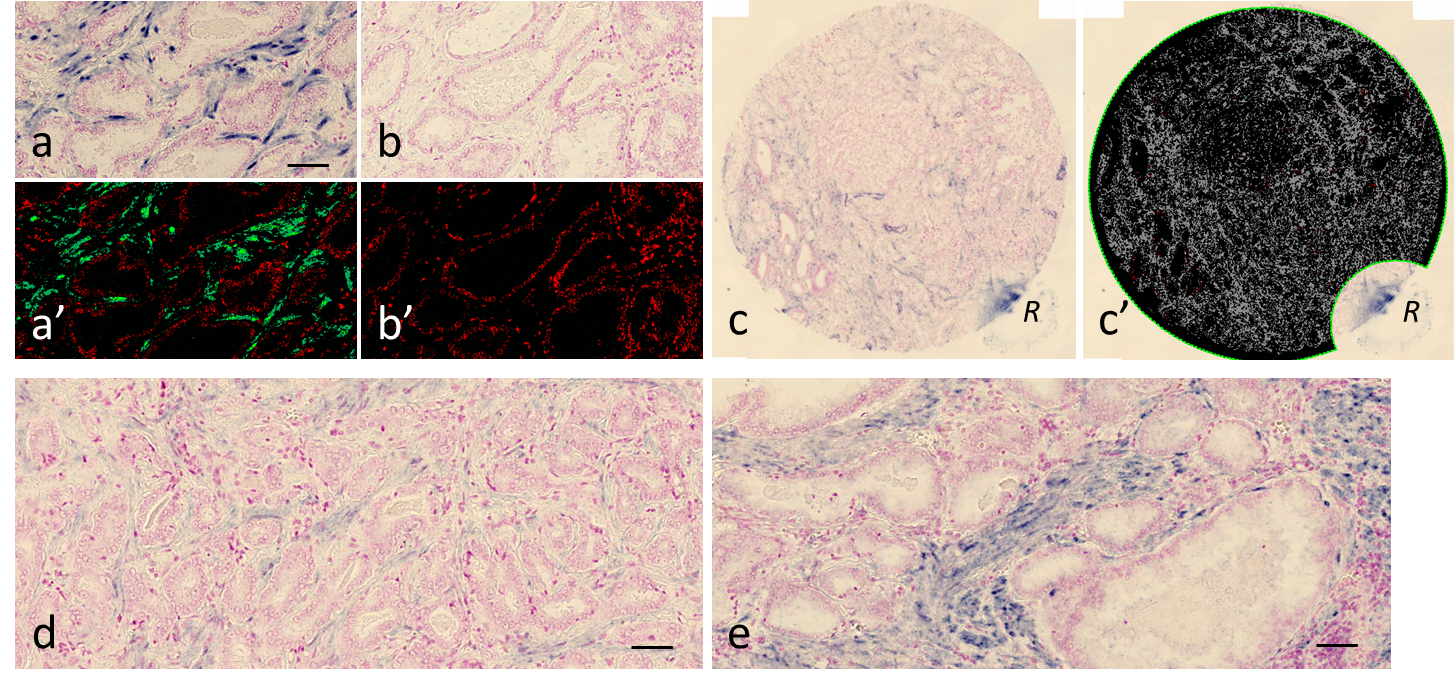
**

**S1 Figure: Quantitation of miRNA by image analysis in prostate cancer**.

Stained slides were processed by image analysis using a trained pixel classifier. Example of ISH signal obtained with probes for miRNA-145 (**a**) and scramble (**b**) without (**a** and **b**) and with pixel classification (**a’** and **b’**). The blue ISH signal and the red counterstain (**a** and **b**) were translated into green and red colors in the classified images (**a’** and **b’**), respectively. Non-specific blue precipitates generated by the staining procedure, here for miRNA-143 (**c**), were removed from the region of interest by Refinement (*R*) that is visible in the classified image (**c’**). Examples of low  (**d**) and high (**e**) expression of miRNA-143. The corresponding relative expression estimates for the patients were 0.04 and 0.11, respectively. Bars: **a** and **b** = 50µm, **d** and **e** = 60µm. The diameter of the TMA core in **c** is 2 mm.
